# Sensor for Distance Estimation Using FFT of Images

**DOI:** 10.3390/s91210434

**Published:** 2009-12-22

**Authors:** José L. Lázaro, Angel E. Cano, Pedro R. Fernández, Carlos A. Luna

**Affiliations:** Electronics Department, University of Alcalá, Superior Polytechnic School, University Campus, Alcalá de Henares (28871), Madrid, Spain; E-Mail: angel.cano@depeca.uah.es (A.E.C.)Telecommunication Department, University of Oriente, Av. de las Américas, SN, Santiago de Cuba (90900), Cuba; E-Mails: pedro.fernandez@depeca.uah.es (P.R.F.); caluna@depeca.uah.es (C.A.L.)

**Keywords:** Fast Fourier Transform, zero-frequency component, distance estimation, infrared, differential method, artificial vision

## Abstract

In this paper, the problem of how to estimate the distance between an infrared emitter diode (IRED) and a camera from pixel grey-level intensities is examined from a practical standpoint. Magnitudes that affect grey level intensity were defined and related to the zero frequency component from the FFT image. A general model was also described and tested for distance estimation over the range from 420 to 800 cm using a differential methodology. Method accuracy is over 3%.

## 1. Introduction

Artificial vision is widely used in robotics applications. One of the most important tasks in this area is robot positioning. Several sensorial systems have been used for this purpose; however, the vision system is one of the most popular positioning systems.

Vision systems are usually modeled geometrically by characterizing the projection of the 3-D scene onto the image plane [1,2]. Previous papers have been presented in the field of IR, in which the authors developed computer programs and methods [3-5]. In [3], a reducing-difference method for non-polarized IR spectra was described, where the IR signal of a five component mixture was reduced stepwise by subtracting the IR signal of other components until total elimination of the non desired signals was achieved. In [4,5], the authors discuss the fundamental questions concerning the morphology of suspended particles and particle size, etc, in chemistry; for this task conventional and linearly polarized IR-spectroscopy were used. Statistical approaches have been developed in order to estimate the impact of the experimental parameters on the IR-signal for each of the several systems studied.

Independently of the kind of projection used to model the vision system, only 2-D robot positioning can be carried out when using a single camera. In other words, depth information is lost if only one camera is used for robot positioning [1,2].

To determine the depth, additional information must be included in the geometrical model. This implies two alternatives: either (a) to use a system with two or more cameras (passive vision or stereo vision) [2-4], or (b) to use a system with a specific illumination pattern (active vision by laser emitter patterns) [5,6].

Consider a local positioning system for an indoor application, with the vision sensor installed in the environment. Mobile robots carry an IRED which is detected by environment cameras. When this situation is analyzed with one camera and the IRED, only one point will be detected, thus 3-D positioning remains an undetermined problem [2]. However, additional information can be extracted from pixel-grey level intensities in order to obtain redundancy for the positioning task, using a geometrical camera model.

The idea of using a single IR point to develop an effective and practical method is based on the fact that the final application will be carried out in settings such as intelligent spaces, smart living, *etc.*, where a certain number of cameras are already distributed.

Later, when the method for distance estimation using FFT has been fully developed, it will be possible to carry out fusion of this data with data obtained from the geometric calibration of cameras in order to improve generation of the variables and parameters involved in pose and location of the mobile incorporating the IRED.

Under the conditions described above, the distance between the IRED and the camera could present a solution to obtaining the third dimension lost in projection based models.

To summarize, this paper presents research carried out in order to develop a sensor based on FFT of images, the contribution, innovations and benefits of which can be summarized as follows:

- the development of a sensor for distance measurement and pose detection based on gray level intensity of the images;
- the distance between two points (IR-camera) can be obtained using a differential method based on zero frequency components from the FFT image;
- the sensor and method are economic, since cameras are already in the application environment (requiring only one IRED for each object/mobile to be detected);
- the method is simple to launch;
- installation of the system is easy;
- the system is noninvasive and safe;
- the sensorial system is complementary to other methods, facilitating ease of data fusion; *etc.*

## 2. Background

When an image is captured, the camera's optical system focuses the light distribution of the scene onto the image sensor, which accumulates this information during the exposure time. An electronic system attached to the image sensor translates the accumulated energy into an electrical signal, which is sampled, quantized and coded. The output of this process comprises discrete values (grey level intensities) for each discrete area (image pixel) of the image sensor Therefore, a relationship exists between the light conditions of the scene and the pixel grey level intensities of images [7,8].

*A priori*, a direct proportionality between grey level intensities, light source intensities, camera exposure time and inverse square distance is assumed. This statement can be obtained if radiometry is applied to model the image formation and the inverse square distance law is taken into account [9].

The light intensities of the source, and distance between the camera and the source constitute magnitudes that affect irradiance on the sensor surface, and thus the output grey level intensity [9].

On other hand, camera exposure time varies the amplitude of the electrical signal converted by the image sensor, because it varies the integration time. Thus, there is a linear relationship between grey level intensities and camera exposure time [7,8].

An analysis using pixel grey level intensities could be performed using a radiometrical approach, to estimate the relationship between IRED radiant intensity *P_e_*, camera exposure time t, and distance between the IRED and the camera *d*. In this case, the inverse camera response function must be estimated [7,8]. However, if light conditions in the scene change the grey-level intensities in images, then they will also change the grey-level distribution in images. Therefore, changes can also be seen on the image spectrum, and a practical characteristic could be extracted from there.

## 3. Characterizing the Camera-IRED Distance Estimation Problem

**Figure 1.**
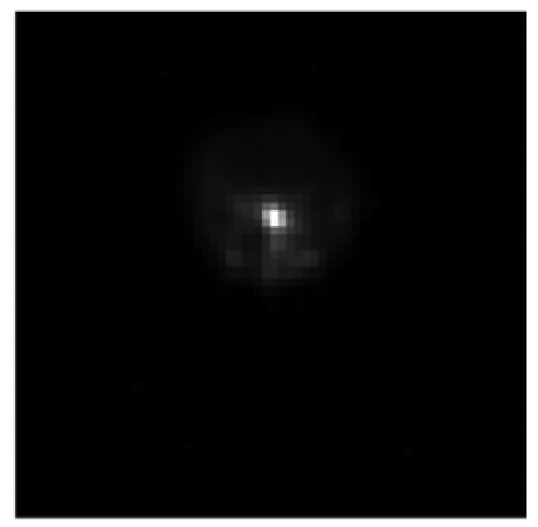
A representative image of the IRED used for the Camera-IRED distance estimation problem.

To characterize the camera-IRED distance estimation problem, several images were captured to find a practical measure which related IRED radiant intensity (*P_e_*), camera exposure time (*t*) and distance between the emitter and the camera (*d*). Figure 1 shows a representative image of the IRED used for the camera-IRED distance problem.

**Figure 2.**
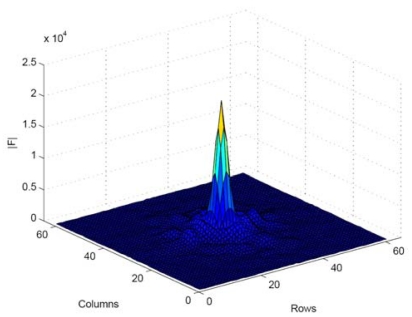
Amplitude spectrum of a representative image of the IRED.

A practical measure related to *P_e_, t* and *d* must be extracted from the captured images. Figure 2 shows the shifted FFT of the image produced by the IRED in the camera. The image spectrum contains all the information about pixel distribution, which is equivalent to possessing the information about scene light conditions. Furthermore, a high percentage of the information concerning pixel distribution in the image of the IRED is contained around zero-frequency.

Thus, we proposed using the zero-frequency component (DC-component: direct current component) as the practical measure to be related to *P_e_, t*, and *d*.

The goal is to obtain a function *f* such that:

(1) $$F(0,0)=f(P_{e},t,d)$$

which means that the DC component *F*(0,0) is a function of *t, P_e_* and *d*.

The function *f* can be estimated from the behavior of *F*(0, 0) with *t, Pe* and *d*, by analyzing different images obtained using different distances between the IRED and the camera, different exposure times and different emitter radiant intensities. The term *F* represents the Fast Fourier Transform of the image. The DC component of the image spectrum gives a measure of mean grey level intensity, which is an indirect measure of the light energy falling on the sensor surface.

### 3.1. Behavior of the DC Component with Camera Exposure Times

**Figure 3.**
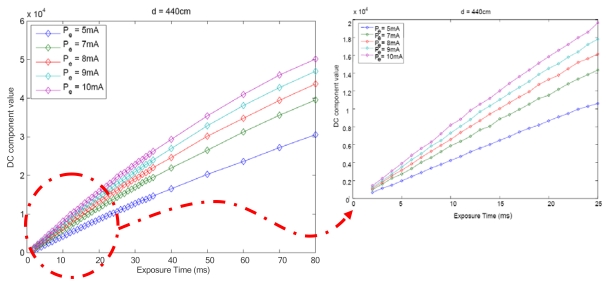
Behavior of the DC component with the camera exposure time for different emitter radiant intensities.

The first experiment to characterize the function *f* was performed varying the camera exposure time. The emitter was placed in front of the camera at a fixed distance and five different IRED radiant intensities were considered. The emitter radiant intensity was adjusted by varying the IRED polarization current. In this experiment, for each polarization current, 38 camera exposure times were used. Results for this experiment are shown in Figure 3, where five different polarization currents were also used. As shown in the Figure 3 this relationship is nonlinear, although it can be approximated with a linear equation for a short exposure time range (up to 20 ms).

Because of the nonlinearity of the relationship between the FFT DC component of IRED image and the camera exposure time, error increases as the exposure time range extends; this explains the experimental results shown in Figure 6. Model calibration was carried out with a range of 4 to 20 ms.

The linear model only works well for a small range of exposure times; however, in practice, this behavior is not important as regards our current applications since the maximum exposure time used was 20 ms as longer exposure times is inefficient in RT applications and could limit the speed of the mobiles incorporating IRED.

### 3.2. Behavior of the DC Component with Ired Polarization Current

A similar experiment was carried out to obtain the behavior of the DC component with emitter radiant intensity or the IRED polarization current. For this experiment, 5, 7, 8, 9 and 10 mA were used and distance between the IRED and the camera was 440 cm.

**Figure 4.**
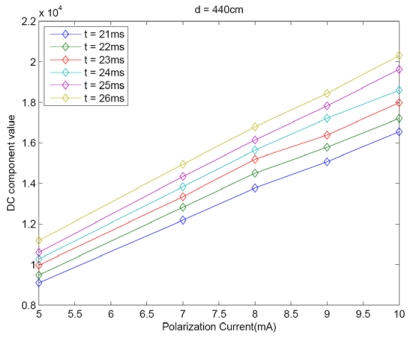
Behavior of the DC component with IRED polarization current for different camera exposure times.

Figure 4 shows that the DC component could be also modeled by a linear function of the IRED polarization current. Only partial results obtained in this experiment are shown in Figure 4, in order to ensure clarity in the Figure, where t from 21 to 26 ms is represented. Nevertheless 38 camera exposure times were used in the experiment.

### 3.3. Behavior of the DC Component in Relation to the Distance Between the IRED and the Camera

**Figure 5.**
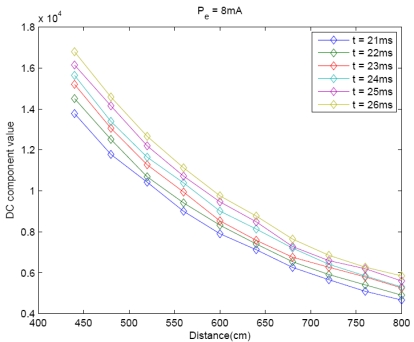
Behavior of the DC component in relation to the distance between the IRED and the camera.

The behavior of the DC component in relation to the distance between the camera and the IRED was also measured. The results are shown in Figure 5. As stated in radiometry, the DC component decreases with square distance. Thus the inverse square distance law could be used in the model.

### 3.4. Integrating DC Component Behaviors into a Model

Figures 3, 4 and 5 are partial results for the experiment to define a model for distance estimation. As Figures 3 and 4 show the DC component of the image spectrum could be modeled by a linear function of *t* and *P_e_*. For each experiment, it can be stated that:

(2) $$F(0,0)=(\tau_{1}t+\tau_{2})K_{d,P_{e}}$$

(3) $$F(0,0)=(\rho_{1}P_{e}+\rho_{2})K_{d,t}$$

However, Figure 5 shows that the DC component decreases as the distance between the camera and the IRED increases. It could be modeled by:

(4) $$F(0,0)=(\frac{\delta_{1}}{d^{2}}+\delta_{2})K_{t,P_{e}}$$

Each of the behaviors shown in Figures 3, 4 and 5 were obtained by setting other parameters; for example, *F*(0, 0)*'s* behavior with camera exposure time was obtained by varying the exposure time and fixing the emitter polarization current and the distance between the IRED and the camera. This meant that other behaviors were evaluated for particular values of d and *P_e_*, which is why the term *K_d_*,*_Pe_*, in (2), is used in the exposure time characterization.

Finally, a global expression for *F*(0, 0) would be as follows:

(5) $$F(0,0)=(\tau_{1}t+\tau_{2})\cdot (\rho_{1}P_{e}+\rho_{2})\cdot (\frac{\delta_{1}}{d^{2}}+\delta_{2})$$

where *τ*_1_ and *τ*_2_ are parameters for modeling the linear behavior of the DC component with camera exposure times, *ρ*_1_ and *ρ*_2_ are used for the behavior of the DC component with IRED radiant intensity and *δ*_1_ and *δ*_2_ model the DC component's behavior with respect to the distance between the IRED and the camera.

By suppressing the parentheses in (5), the DC component would yield:

(6) $$F(0,0)=k_{1}\frac{P_{e}t}{d^{2}}+k_{2}\frac{P_{e}}{d^{2}}+k_{3}\frac{t}{d^{2}}+\frac{k_{4}}{d^{2}}+k_{5}P_{e}t+k_{6}t+k_{7}P_{e}+k_{8}$$

where *κ_i_* with *i* = 1, 2,*…*, 8 are parameters of the model that must be calculated in a calibration process (see Section 4.1.).

### 3.5. Estimating the Distance between the IRED and the Camera

Once values for parameters κ_í_ are obtained, a differential methodology can be applied for distance estimation. Differential methodology reduces the number of parameters necessary for the distance estimation process, and also reduces the effect of the offset illumination in the scene. Therefore, a more robust and efficient methodology can be applied.

If two images of the IRED are captured whilst varying the camera exposure time and considering that the emitter is static at a fixed distance from the camera, the difference between extracted DC components can be written as:

(7) $$F_{2}(0,0)-F_{1}(0,0)=k_{1}\frac{P_{e}(t_{2}-t_{1})}{d^{2}}+k_{3}\frac{\left(t_{2}-t_{1}\right)}{d^{2}}+k_{5}P_{e}(t_{2}-t_{1})+k_{6}(t_{2}-t_{1})$$

with *F_1_*(0, 0) and *F_2_*(0, 0) the DC component of the image captured with t_1_ and t_2_, respectively. Thus, solving (7) for distance:

(8) $$d=\sqrt{\frac{k_{1}P_{e}(t_{2}-t_{1})+k_{3}(t_{2}-t_{1})}{F_{2}(0,0)-F_{1}(0,0)-k_{5}P_{e}(t_{2}-t_{1})-k_{6}(t_{2}-t_{1})}}$$

In practice, *t_1_* is used as a reference camera exposure time and a few images are captured in order to apply the differential method. As a general approach, *t_2_* is substituted by *t_j_, F_2_*(0, 0) by *F_j_*(0, 0) with *j* = 1, 2,…,*N* where *N* is the total of exposure times considered in the estimation process. Thus, *N* distance estimations would be obtained. The final result for distance between the IRED and the camera is the average of those *N* estimations.

## 4. Practical Implementation of the Method for Distance Estimation

The practical implementation of estimating the distance between an IRED and a camera uses an SFH4200Z IRED with a typical wavelength emission of 940 nm. The camera is a Basler A622f, with an interference filter attached to the optics in order to eliminate the effect of background illumination. To use the model proposed in (6) in a differential form, Equations (7) and (8), and a calibration process are required to obtain values for *k_i_*.

### 4.1. Calibration Process

To implement the calibration process, images were captured using a distance of 440, 560, 720 and 800 cm between the IRED and the camera. In addition, for calibration data, five IRED radiant intensities, corresponding to five different polarization currents (5, 7, 8, 9 and 10 mA) and a range of exposure times from 2 to 21 ms, with a step of 1 ms, were used. The image sequence was created by considering five polarization currents for each distance, with 20 camera exposure times for each polarization current. A total of 500 images were used for calibration data.

In practice, a selection of over 21 ms for camera exposure time is inefficient since it increases the limit for mobile speed. However, this research was not limited to this exposure time restriction, additionally investigating longer exposure times.

Values for *K_i_* coefficients were calculated by minimizing the mean square error *ε* from:

(9) $$ɛ={\left|F(0,0)-(k_{1}\frac{P_{e}t}{d^{2}}+k_{2}\frac{P_{e}}{d^{2}}+k_{3}\frac{t}{d^{2}}+\frac{k_{4}}{d^{2}}+k_{5}P_{e}t+k_{6}t+k_{7}P_{e}+k_{8})\right|}^{2}$$

by solving the system formed by: *A·K*=*F*_0_ where K = [*k*1, *k*2,*…*,*k*8]. A is formed by the combination of *P_e_, t* and *d*; and F_0_ is a vector of DC components.

Model parameters are obtained by:

(10) $$\text{K}={\left(\text{A}\cdot {\text{A}}^{\text{T}}\right)}^{-1}\cdot {\text{A}}^{\text{T}}\cdot {\text{F}}_{0}$$

using much data to solve an over-determined Equation system.

**Figure 6.**
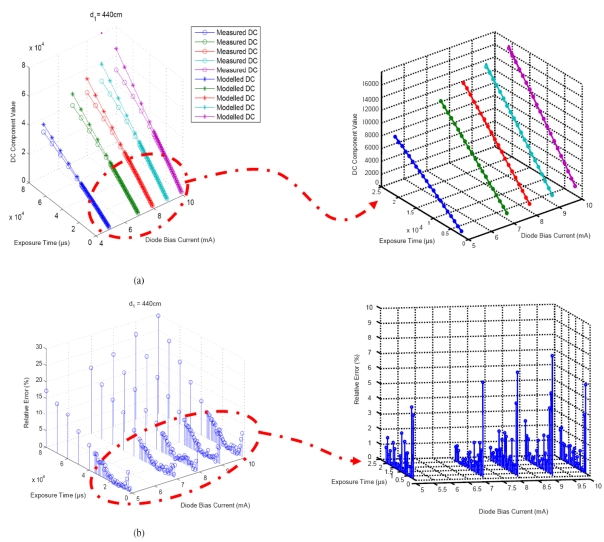
Results for the calibration process. (a) Model performance. (b) Relative error in the calibration process.

Figure 6 shows the result for the calibration process and its corresponding relative error. Once the calibration process has been performed, the distance can be estimated using the differential method. In Figure 6-b it can be seen that the errors are consistently and significantly greater for short exposure times near 0 ms.

The reason for this may be that the relative accuracy of the camera in determining exposure times is worse for shorter times (an equal absolute error produces a higher relative error in energy capture); however, it may also be due to imperfections in the initial response of the pixels to low irradiance levels and integration of the same, which for very short exposure times are not masked by the signal captured

### 4.2. Distance Estimation by Differential Method

As is explained in Section 3.4, the expression (8) can be used for distance estimation. The distance estimation process experiment was carried out in two stages. First, images were taken using distances of 440 to 800 cm with a 40 cm step, which were considered as the real distance between the IRED and the camera. The second part of the experiment used distance values in the middle of the interval used in the first stage; that is, distances from 420 to 740 cm with a step of 40 cm were considered. The image sequences used in both experiments were captured in different weeks; however both distance ranges were treated as one.

**Figure 7.**
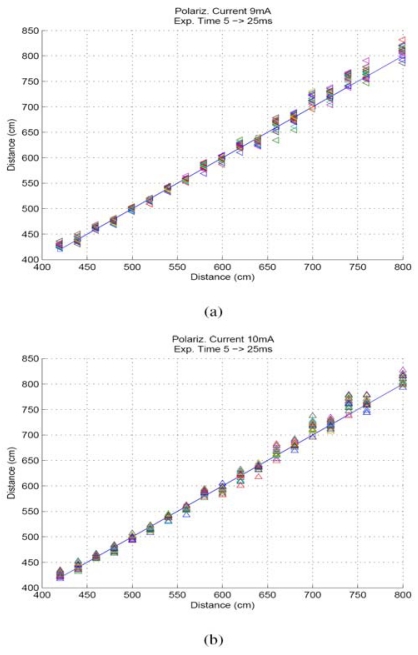
Distance estimation for different exposure times from 5 to 25 ms. (a) Polarization current 9. mA. (b) 10 mA.

In Figure 7, the distance estimation corresponding to 5, 6,…,25 ms, with 2 ms used as reference exposure time, is shown. The polarization currents used in these results are 9 and 10 mA respectively. For other polarization currents considered in the calibration process, similar results were obtained. In Figure 6, 20 distance estimations were originally shown, which is inefficient in practice. Thus, it was necessary to make a selection from different camera exposure times, given that the reference exposure time was already fixed.

Thus, camera exposure times from 12 to 17 ms, with 2 ms as reference camera exposure time, were selected to estimate the distance. Using this range of exposure times, five distance estimations were calculated and averaged to obtain the final distance estimation.

## 5. Results

Tables 1 and 2 show the results for final distance estimation using the differential method for 7, 8, 9 and 10 mA of polarization current.

## 6. Conclusions

This paper presents an analysis of the estimation of the distance between a camera and an infrared emitter diode. The aim was to provide an alternative for estimating the depth which is lost when the camera's geometrical model is applied for positioning purposes.

A model using a characteristic extracted from grey-level intensities was defined from an empirical point of view, to obtain a relationship with the distance between a camera and an IRED. To define the model, several images were captured under different conditions, to ensure that all possible combinations were modeled.

The research took into account the magnitudes that affect grey-level intensities in images and related them to the zero-frequency component extracted from the image's FFT. The results of these experiments demonstrate that the DC component, for a small range of exposure times, is a linear function of camera exposure time, IRED radiant intensity (represented by the IRED polarization current) and the inverse square distance. These relationships were measured independently by DC component behaviors and were also integrated into one expression.

The distance was also estimated by a differential method, which ensured a robust performance and an efficient methodology. Two images captured with different camera exposure times are required to apply the differential method. One of the images is used as reference image. The methodology was generalized for more than two camera exposure times.

In the paper we demonstrated that by using images captured with six different exposure times; arranged in two ranges; such as: from 4 to 9 ms and from 12 to 17 ms, with 2 ms as reference exposure time; the distance between an IRED and a camera can be estimated accurately. Obtained results showed that the accuracy of the proposal is lower than 3% over the range from 420 to 800 cm of distances between the IRED and the camera.

An important point that we would like to highlight is that two experiments were carried out in different weeks using the same model parameters. Tests were performed on different days with different lighting conditions and background noise. Note that, as was indicated in Section 4 (Results), an interference filter attached to the optics in order to eliminate the effects of background illumination was used.

Tests were carried out in corridors with no natural lighting (no windows), with and without artificial light. This gave similar responses, as was to be expected by introducing a 10 nm bandwidth interference filter.

Tests were also carried out in a room with large windows (and therefore with the potential influence of the sun) on different days and at different times; artificial lighting noise was also introduced at times. Some measures were even taken with the camera in front of the windows, with no significant differences obtained in measures. Note that in addition to using an interference filter, a very high power IRED was used and camera exposure times were short. Those parameters were calculated from a sample of images from the first experiment; however, different values for distance, within the range of 400 to 800 cm, were used in the second experiment. Nevertheless, equivalent accuracy was obtained.

## Figures and Tables

**Table 1.** Distance Estimation for each polarization current.

**Real Distance(cm)**	**Est.Dist. (cm) *Pe* = 7 mA**	**Est.Dist. (cm) (*Pe* = 8 mA)**	**Est.Dist. (cm) (*Pe* = 9 mA)**	**Est.Dist. (cm) (*Pe* = 10 mA)**
440	438.2	433.0	435.0	437.4
480	473.6	473.3	470.5	471.0
520	513.9	514.0	515.5	513.9
560	552.8	553.6	554.8	554.0
600	595.3	589.8	595.4	592.5
640	633.8	627.0	628.6	636.3
680	679.7	685.1	675.5	683.6
720	724.3	706.6	721.1	717.5
760	754.9	754.8	764.4	758.5
800	803.2	806.9	810.9	799.2

**Table 2.** Distance Estimation for each polarization current.

**Real Distance(cm)**	**Relat. Error. (%) *Pe* = 7 mA**	**Relat. Error. (%) (*Pe* = 8 mA)**	**Relat. Error. (%) (*Pe* = 9 mA)**	**Relat. Error. (%) (*Pe* = 10 mA)**
440	0.4	1.6	1.1	0.6
480	1.3	1.4	2.0	1.9
520	1.2	1.2	0.9	1.2
560	1.3	1.1	1.0	1.1
600	0.8	1.7	0.8	1.2
640	1.0	2.0	1.8	0.6
680	0.1	0.8	0.7	0.5
720	0.6	1.9	0.2	0.3
760	0.7	0.7	0.6	0.2
800	0.4	0.9	1.4	0.1

**Table 3.** Relative error on distance estimation for the first experiment.

**Real Distance(cm)**	**Est.Dist. (cm)*P_e_* = 7 mA**	**Est.Dist. (cm) (*P_e_* = 8 mA)**	**Est.Dist. (cm) (*P_e_* = 9 mA)**	**Est.Dist. (cm) (*P_e_* = 10 mA)**
420	429.1	424.7	426.0	423.4
460	461.5	460.9	461.5	458.5
500	499.3	497.1	500.2	498.7
540	540.9	542.8	539.9	538.7
580	586.2	586.2	582.5	585.8
620	618.4	628.0	623.6	619.7
660	663.6	663.2	662.7	667.4
700	713.8	708.5	707.6	714.0
740	745.8	754.4	759.2	758.4

**Table 4.** Relative error on distance estimation for the second experiment.

**Real Distance(cm)**	**Relat. Error. (%) *P_e_* = 7 mA**	**Relat. Error. (%) (*P_e_* = 8 mA)**	**Relat. Error. (%) (*P_e_* = 9 mA)**	**Relat. Error. (%) (*P_e_* = 10 mA)**
420	2.2	1.1	1.4	0.9
460	0.3	0.2	0.3	0.3
500	0.1	0.6	0.1	0.3
540	0.2	0.5	0.1	0.2
580	1.0	1.0	0.4	1.0
620	0.3	1.3	0.6	0.1
660	0.6	0.5	0.4	1.1
700	2.0	1.2	1.1	2.0
740	0.8	2.0	2.1	2.5
